# N-acetylcysteine: evidence based consensus document on the therapeutic advantages in respiratory diseases (NECTAR)

**DOI:** 10.3389/fmed.2026.1810363

**Published:** 2026-05-04

**Authors:** Monica Barne, Madhuragauri Shevade, Parthasarathi Bhattacharyya, Devasahayam J. Christopher, Sanjeev Nair, Nitin Abhyankar, Indranil Halder, Agam Vora, Arjun Khanna, Rajesh Swarnakar, Prashant Chhajed, Balamurugan Santhalingam, Aishwarya Nair, Sundeep Salvi

**Affiliations:** 1 Chest Research and Training Pvt. Ltd., Pune, India; 2 Institute of Pulmocare and Research, Kolkata, India; 3 Christian Medical College Vellore, Vellore, India; 4 Government Medical College, Thiruvananthapuram, India; 5 Poona Hospital and Research Center, Pune, India; 6 College of Medicine and JNM Hospital, West Bengal University of Health Sciences, Kalyani, India; 7 BSES Brahma Kumaris’ Global Hospital & Vora Clinic, Mumbai, India; 8 School of Medicine, Amrita Vishwa Vidyapeetham, Faridabad, India; 9 Getwell Hospital and Research Institute, Nagpur, India; 10 Nanavati Max Super Speciality Hospital and Lilavati Hospital, Mumbai, India; 11 ACS Medical College and Hospital, Dr. M.G.R. Educational and Research Institute University, Chennai, India

**Keywords:** antioxidant, bronchiectasis, COPD, cystic fibrosis, mucolytic, N-acetylcysteine

## Abstract

**Background:**

N-acetylcysteine (NAC) is a key precursor of glutathione (GSH), the lung’s principal antioxidant. First developed as a mucolytic, NAC is now recognized for broader antioxidant, anti-inflammatory, immunomodulatory, and anti-biofilm effects, prompting its use as an adjuvant in treatment of chronic respiratory conditions. This document consolidates existing evidence to this effect, and adds insights from practicing clinicians to guide about use of NAC in clinical practice.

**Methods:**

An expert working group (EWG) comprising ten pulmonologists and a multidisciplinary drafting committee reviewed data from *in vitro*, mechanistic, animal and observational studies, randomized trials, reviews and meta-analyses for role of NAC in respiratory diseases. Eight respiratory conditions: stable COPD, acute exacerbations of COPD (AECOPD), tuberculosis (TB), and anti-tubercular drug–induced liver injury (AT-DILI), non-cystic fibrosis bronchiectasis, cystic fibrosis (CF), bacterial and viral infections, interstitial lung disease (ILD), and asthma were studied and discussed. A modified Delphi process was conducted to establish expert consensus on the role of NAC in each of these conditions. Consensus was defined as ≥70% agreement on predefined statements.

**Results:**

Experts agreed that NAC is useful as an adjunct in COPD especially chronic bronchitis phenotypes mainly for preventing exacerbations at 600 mg twice daily. During AECOPD, low dose NAC may aid recovery. In TB and AT-DILI, NAC is safe, lowers oxidative stress and may limit lung damage. In non-CF bronchiectasis, NAC may reduce exacerbations in frequent exacerbators. In CF, NAC improves lung function, mucociliary clearance, and disrupts biofilms. NAC is useful in bacterial and viral infections but data for ILDs and asthma does not support routine use. NAC was consistently rated safe and well-tolerated.

**Conclusion:**

This consensus underscores the role of NAC in chronic respiratory diseases beyond its mucolytic properties and reiterates that NAC’s antioxidant, anti-inflammatory, immunomodulatory and anti-biofilm properties provide significant clinical utility. It is a proven adjunctive therapy for COPD, bronchiectasis, and cystic fibrosis. While promising for TB and respiratory infections, further evidence is required. Its efficacy in asthma and ILDs remains uncertain. These findings guide clinical practice while highlighting research priorities to fully establish NAC’s therapeutic potential in respiratory medicine.

## 1 Introduction

**FIGURE 1:**
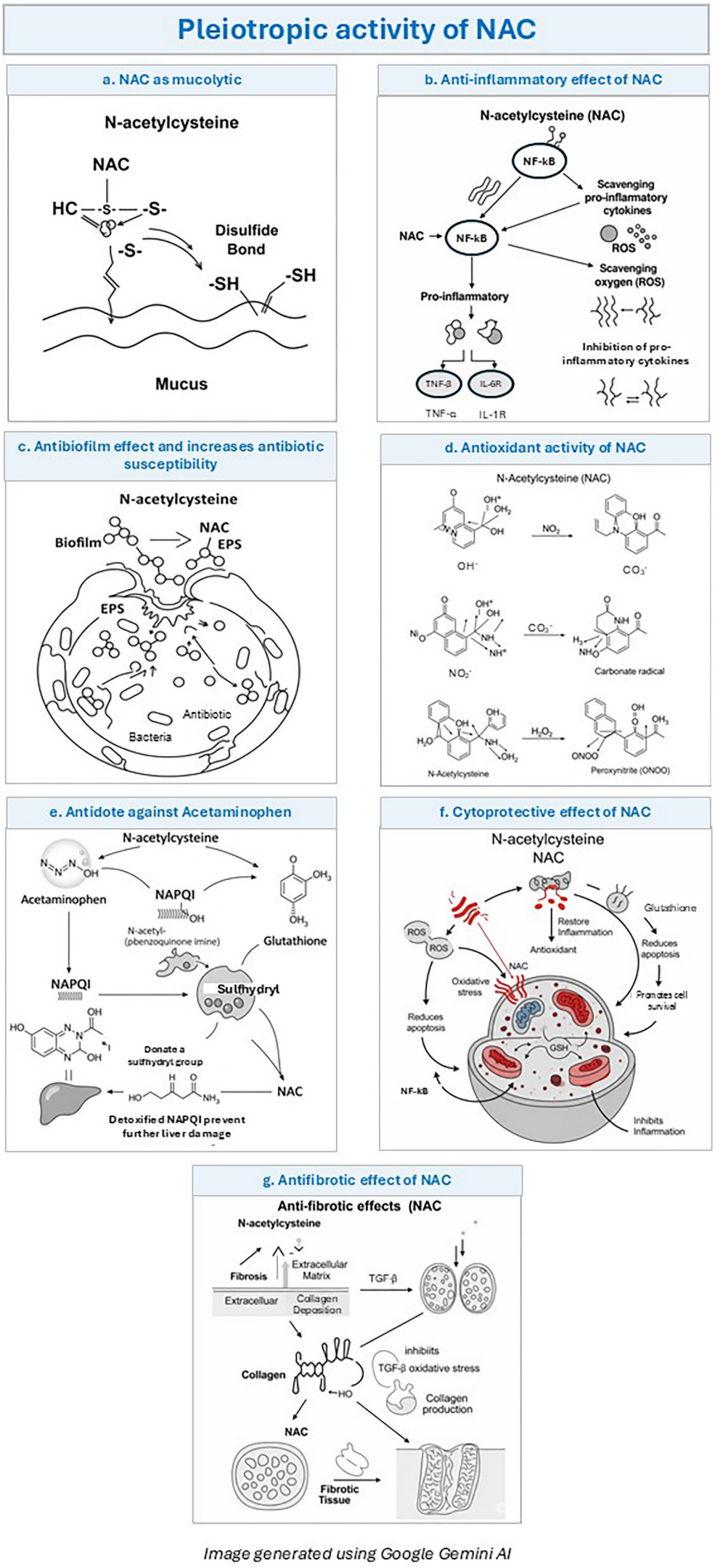
Pleiotropic effect N-acetylcysteine (NAC). **(a)** NAC as mucolytic: N-acetylcysteine (NAC) breaks disulfide bonds within mucin glycoproteins via its sulfhydryl (–SH) group. This reduces mucus viscosity and facilitates improved mucociliary clearance. **(b)** Anti-inflammatory effect of NAC: NAC modulates NF-κB signaling and scavenges reactive oxygen species (ROS), reducing inflammatory activation. This leads to decreased production of pro-inflammatory cytokines such as TNF-α and IL-6. **(c)** Antibiofilm effect and increased antibiotic susceptibility: NAC disrupts biofilm structure by degrading extracellular polymeric substances (EPS). This enhances antibiotic penetration and improves bacterial susceptibility. **(d)** Antioxidant activity of NAC: NAC directly neutralizes reactive oxygen and nitrogen species, including free radicals. It also replenishes intracellular glutathione, strengthening cellular antioxidant defense. **(e)** Antidote against acetaminophen toxicity: NAC replenishes glutathione stores and provides sulfhydryl groups to detoxify NAPQI. This prevents hepatocellular injury and limits liver damage following overdose. **(f)** Cytoprotective effect of NAC: NAC reduces oxidative stress and apoptosis by restoring redox balance and glutathione levels. It inhibits inflammatory pathways and promotes overall cellular survival. **(g)** Antifibrotic effect of NAC: NAC attenuates fibrosis by inhibiting TGF-β signaling and reducing oxidative stress. This decreases collagen deposition and extracellular matrix accumulation in tissues.

N-acetylcysteine (NAC), an acetylated derivative of L-cysteine, serves as a precursor of glutathione (GSH), a key pulmonary antioxidant. Initially developed in the 1960s as a mucolytic agent (1), NAC was later identified as an effective antidote for acetaminophen toxicity by detoxifying N-acetyl-p-benzoquinone imine (NAPQI) (2) (Figure 1). It enhances intracellular GSH synthesis and acts as both a direct and indirect antioxidant, scavenging major oxidants such as OH, NO_2_, and thiyl radicals, though less effectively against superoxide and hydrogen peroxide (3). NAC replenishes depleted GSH levels in Human immunodeficiency virus (HIV) patients (4, 5), prevents contrast-induced nephropathy (6), and has demonstrated therapeutic potential in psychiatric disorders, neurodegenerative diseases, infertility, cancer, ophthalmic, and gastrointestinal conditions (7–12).

Given GSH’s critical antioxidant role in the lungs, NAC has been extensively evaluated in respiratory diseases. By disrupting disulfide bonds in mucin, it provides potent mucolytic action in COPD, cystic fibrosis, and bronchiectasis, showing synergistic effects with short-acting antimuscarinics (13). It penetrates bacterial biofilms, enhances antibiotic sensitivity, and improves mucociliary clearance by stimulating ciliary activity. Its anti-inflammatory, and immunomodulatory properties further strengthen its therapeutic relevance in chronic respiratory disorders (14). Despite robust evidence, NAC remains underutilized; greater clinical integration requires clearer guideline recommendations and clinician awareness. This review consolidates current evidence and expert consensus on NAC for clinical reference.

## 2 Expert consensus on NAC’s role in respiratory conditions

N-acetylcysteine has been utilized by clinicians for many years to manage different respiratory conditions, with varied experiences and outcomes. We aimed to develop this document based on the above understanding.

## 3 Methodology

A modified Delphi exercise was conducted to achieve expert consensus. The expert working group panel consisted of ten pulmonologists with extensive clinical experience in managing chronic respiratory diseases. The drafting committee comprised a pulmonologist, a primary care physician, a doctoral-level respiratory therapist, and a research scientist. The expert panel and drafting committee convened virtually on three occasions. During the initial meeting, the premise was identified and the overall methodology was defined. It was subsequently agreed that the consensus statement would address the role of oral NAC across, eight common respiratory conditions, namely: stable COPD to prevent exacerbations, during an acute exacerbation of COPD (AECOPD), tuberculosis (TB) and anti-tubercular drug-induced liver injury (AT-DILI), non-cystic fibrosis bronchiectasis, cystic fibrosis, lung infections, interstitial lung disease (ILD), and asthma.

An extensive literature search was conducted by the drafting committee using PubMed, Google Scholar and Google employing the search terms, “N-acetylcysteine” or “NAC” in combination with any of the following disease-specific keywords including, “chronic respiratory diseases,” “chronic bronchitis,” “chronic obstructive pulmonary disease,” “COPD,” “exacerbation of COPD,” “AECOPD,” “prevention of AECOPD,” “management of AECOPD,” “acute exacerbation of COPD,” “tuberculosis,” “TB,” “anti-tubercular treatment,” “liver toxicity,” “hepatotoxicity,” “cystic fibrosis,” “non-cystic fibrosis bronchiectasis,” “bronchiectasis,” “interstitial lung disease,” “asthma,” “hyper-reactive airways,” “Interstitial Lung Disease,” “ILD,” “diffuse lung disease,” “pulmonary fibrosis,” “idiopathic pulmonary fibrosis,” “atypical pneumonitis,” “chronic ’hypersensitivity pneumonitis,” “sarcoidosis,” “bacterial infections,” “viral infections” and “COVID-19.”

For each of the eight disease conditions, every reference paper were reviewed and summarized according to study design {[*in vitro*, animal study, randomized controlled trial (RCT), review, meta-analysis, etc.], sample size, methodology results and key outcomes}. The summaries were compiled in both narrative and tabulated formats and circulated to the expert panel for review. The compiled evidence was discussed during the second virtual meeting, and additional references were incorporated based on panel recommendations. Following this review, a set of disease-specific consensus statements was developed and distributed to the expert panel for a structured evaluation. The panelists were asked to indicate their level of agreement for each statement by selecting one of the six response options: “strongly agree,” “mostly agree,” “agree,” “mostly disagree,” “completely disagree,” “not answered” based on their knowledge and experience.

The panel responses to the consensus statements were synthesized into recommendations. Consensus in favor of a statement was predefined as ≥70% of the respondents, with agreement categorized as “strongly agree,” “mostly agree,” or “agree” (15).

A third virtual meeting was subsequently convened to review the draft recommendations; the statements were either accepted, rejected or modified based on collective panel discussion and final consensus.

The resulting consensus provides an integrated synthesis of available evidence, incorporating data from RCTs, systematic reviews, meta-analyses, *in vitro*, and animal studies. Aggregated panel responses formed the basis of the final recommendations, which are intended to provide clinicians an experience-driven guidance for practical application.

## 4 Role of NAC in stable COPD for prevention of exacerbation

India suffers a high burden of COPD. The estimated pooled prevalence of COPD in India is 11.1% (95% CI: 3.5–18.7) (16) and India has the highest number of deaths due to COPD in the world. Mortality is associated with COPD exacerbation and a key goal of management of stable COPD is prevention of an AECOPD. This is important because exacerbations are significant events in patients with COPD. They adversely impact life by causing hospitalization, irreversible loss of lung function, increasing risk of future exacerbation, mortality, and a huge economic cost (17). NAC has been found to be useful in prevention of exacerbations in COPD due to its mucolytic, antioxidant, anti-inflammatory and anti-infective activities (14). Newer evidence suggests that presence of mucus plugs increases all-cause mortality in COPD patients even in Global Initiative for Chronic Obstructive Lung Disease (GOLD) Stage 1 and proposes an early role for thiol based mucolytics for better disease outcomes (18, 19).

### 4.1 Summary of randomized controlled trials (RCTs)

Prospective, multicentric, RCTs have demonstrated that NAC offers multiple clinical benefits in COPD management. These include symptomatic improvement, notably in sputum characteristics, i.e., it reduces viscosity and volume (20, 21) alleviates cough (22), and significantly decreases exacerbation frequency (21, 23–25). NAC also exerts antibiotic-sparing effects (21), reduces hospitalization rates (25), and enhances lung function parameters such as Forced Expiratory Volume in 1 Second (FEV_1_) and Peak Expiratory Flow Rate (PEFR) (25). Additionally, NAC reduces sick leave days (23, 24, 26) and improves overall quality of life (24). Reduction in exacerbations due to NAC also has a dose response effect and the risk of hospital readmissions is seen to reduce gradually and significantly with increasing doses of NAC (27).

Initial studies utilizing low-dose NAC (600 mg/day) yielded promising results, prompting the initiation of larger multicenter RCTs focused on GOLD-defined COPD patients. Notably, two such trials with low dose NAC, 600 mg/day over 3 years demonstrated some efficacy in inhaled corticosteroid (ICS)-naïve patients only (28, 29).

High-dose NAC (1,200 mg/day) showed superior outcomes, improving lung volumes [Forced Vital Capacity (FVC), Residual Volume/ Total Lung Capacity (RV/TLC), Inspiratory Capacity (IC)] and exercise tolerance(30). As poor lung function independently increases COPD exacerbation risk (31), these findings are clinically relevant. In the HIACE RCT, Tse et al. reported that NAC (1,200 mg/day) improved Forced Expiratory Flowrate at 25%–75% (FEF_25–75%_), reactance at 6 Hz, and resonant frequency over 1 year, while these declined with placebo (32). The HIACE trial and post hoc analyses also showed reduced exacerbation rates and prolonged time to first exacerbation, particularly in GOLD C and D categories (33).

The PANTHEON trial, a large double-blind RCT in GOLD defined COPD patients with severe airflow obstruction and a history of ≥2 COPD exacerbations in the previous year, found that NAC 600 mg BID reduced exacerbation rates in NAC group (497 acute exacerbations in 482 patients, 1.16 AcEx/pt/yr) versus placebo (641 acute exacerbations in 482 patients, 1.49 AcEx/Pt/yr), *p* < 0.05 (34). Mean duration of exacerbation was also lesser in the NAC group (14.8 ± 13.9 days) as compared to placebo (19.2 ± 21.1 days) (*p* = 0.003). The effect was more pronounced in moderate COPD GOLD Stage II than severe GOLD Stage III disease. An extensive *post hoc* subgroup analysis of the PANTHEON (35) showed a significant (23%, *p* = 0.01) reduction in exacerbation in smokers and ex-smokers in the NAC group versus the placebo. Further analysis based on the medication history showed that NAC worked best in COPD patients who were not taking inhaled corticosteroids (ICS). In patients without ICS, NAC reduced all exacerbations by 27% compared to placebo. When combined with long-acting bronchodilators (but still without ICS), NAC reduced all exacerbations by 49% as compared to LABA + Placebo and reduced moderate-to-severe exacerbations by 57%. Further analysis showed NAC plus bronchodilators (without ICS) was much more effective than bronchodilators plus ICS and reduced overall exacerbations by 60%, moderate exacerbations by 64%, and severe exacerbations by 88%. This indicates that NAC + LABA may be a safer alternative for frequent exacerbators than adding ICS to LABD.

While 1,200 mg/day NAC consistently improved symptoms, quality of life, exercise capacity, and biomarkers of oxidative stress (36, 37), a recent RCT indicated a significant decrease in the annual rate of moderate/severe exacerbations which were 24% lower in the NAC group than in the placebo group (0.34 vs. 0.45 per patient-year; RR, 0.76; 95% CI, 0.64–0.90; *P* = 0.001), while non-statistically significant trend towards reduced total exacerbations (38).

### 4.2 Summary of meta-analyses and reviews

Meta-analyses and reviews have refined understanding of NAC’s role in stable COPD. The PANTHEON study was initially criticized by Cazzola et al. (39), Turner et al. (40) stating that the results may not be applicable to all COPD phenotypes, perhaps not be replicable globally as the study population was limited to China and that the results are probably due to the mucolytic effect of NAC and not due to the anti-inflammatory or antioxidant properties. However, Cazzola et al. later published a meta-analysis of 13 studies (4,155 COPD patients; NAC *n* = 1,933, controls *n* = 2,222) and found that NAC significantly and consistently reduced exacerbations of COPD 25% reduced risk (CI 16%–34% *p* < 0.01) as compared to controls or placebos. They also found that 1,200 mg per day was required for patients with documented airflow obstruction while patients with only symptoms may benefit with 600 mg per day (41). These findings align with prior reviews (42–45) and Cochrane analyses confirming NAC’s efficacy in exacerbation prevention. The two Cochrane reviews, including 26 studies (6,233 participants) and 28 studies (6,723 participants), reported that compared to placebo, NAC significantly increased the likelihood of patients remaining exacerbation-free (OR 1.75, 95% CI 1.57–1.94; and OR 1.73, 95% CI 1.56–1.91, respectively) (46, 47).

While Huang et al. found no significant reduction in exacerbation risk or lung function decline, likely due to study heterogeneity (48), a recent systematic review of 20 studies (4,044 patients) by Papi et al. reported a 24% reduction in exacerbations and improved symptoms and quality of life in pre-COPD patients (49). A narrative review by the same group suggested that NAC’s sputum-modifying effect may be beneficial even in early COPD (50).

Overall, evidence indicates that high-dose NAC (1,200 mg/day) reduces exacerbations, improves lung function, and enhances quality of life, particularly in ICS-naïve patients or those on bronchodilators. Large RCTs and meta-analyses collectively support NAC’s mucolytic, antioxidant, anti-inflammatory, and anti-infective benefits in COPD.

In summary,

1. N-acetylcysteine reduces exacerbations consistently across multiple meta-analyses and Cochrane reviews (∼24%–25% reduction).
2. Improves symptom control and quality of life, including in early/pre-COPD patients.
3. Dose matters:
  a. 1,200 mg/day → patients with airflow obstruction.
  b. 600 mg/day → milder or symptomatic patients.
4. Benefits are likely driven primarily by mucolytic effects, with possible additional antioxidant/anti-inflammatory roles.
5. Evidence is strong but somewhat heterogeneous, with a few studies showing neutral results.
6. Useful as an adjunct therapy in long-term COPD management, especially for exacerbation prevention.

### 4.3 Recommendations by European Respiratory Society/American Thoracic Society (ERS/ATS) 2017 and GOLD 2025 guidelines

ERS/ATS recommendation 2017 (51): “For patients who have COPD with moderate or severe airflow obstruction and exacerbations despite optimal inhaled therapy, we suggest treatment with an oral mucolytic agent to prevent future exacerbations.”

GOLD 2025 (52): “In COPD patients not receiving ICS, regular treatment with mucolytics such as carbocysteine and NAC may reduce exacerbations and modestly improve health status.”

### 4.4 Consensus of panel of experts

**FIGURE 2:**
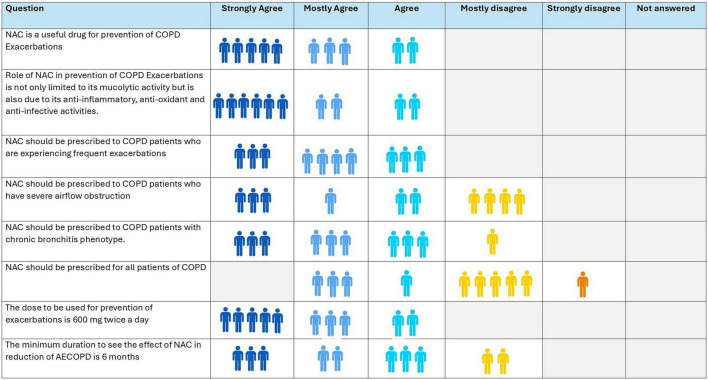
Opinion of experts on role of N-acetylcysteine (NAC) in stable chronic obstructive pulmonary disease (COPD) for prevention of acute exacerbation.

The expert panel agreed that in management of stable COPD, NAC is beneficial for prevention of AECOPD owing to its mucolytic, anti-inflammatory, antioxidant, and anti-infective properties (Figure 2). NAC was recommended particularly for patients with frequent exacerbations. Ninety percent supported its use in patients with the chronic bronchitis phenotype. While 60% agreed that it should be prescribed for severe airflow obstruction, only 40% recommended prescribing NAC to all COPD patients. During the third virtual meeting, the experts were then asked to opine on the GOLD stage of COPD that they would most commonly prescribe NAC. There was a unanimous and strong agreement in favor of its use in GOLD stage E patients, with 50% supporting its use in GOLD stage B and 25% in GOLD stage A patients. The consensus-recommended dose was 600 mg BID, with optimal clinical benefit observed after 6 months’ therapy.

**Recommendation:**

1. N-acetylcysteine is indicated for prevention of AECOPD in patients with stable COPD, particularly those with chronic bronchitis phenotype characterized by mucus hypersecretion, frequent exacerbations, severe airflow limitation, and significant mucus accumulation (53).
2. GOLD stage E patients should receive NAC routinely, GOLD stage B patients may be considered on a case-by-case basis, and stage A patients are generally not indicated.
3. Standard dosing is 1,200 mg per day (600 mg twice daily), with a minimum duration of 6 months for optimal effect.

## 5 Role of NAC during ongoing AECOPDs

The *in-vitro* studies have shown that COPD exacerbations are characterized by increased inflammatory cellular infiltration, upregulation of cytokines, chemokines, adhesion molecules as well as increased levels of oxidative stress markers like H_2_O_2_, 8-isoprostane and depletion of GSH (54). Cazzola et al. demonstrated the beneficial effect of NAC in an *ex vivo* lipopolysaccharide (LPS) model of human tracheal rings, showing a reduction in oxidative markers such as peroxidase activity, H_2_O_2_, malondialdehyde and nitric oxide, alongside an increase in antioxidant levels including GSH and superoxide dismutase, as well as inhibition of inflammatory responses (55).

### 5.1 Summary of RCTs

The anti-inflammatory and antioxidant effects of NAC were demonstrated in two RCTs showing significant clinical benefits. In a 123-patient, three-arm study (NAC 1,200 mg, 600 mg, placebo), NAC 1,200 mg was superior to 600 mg and placebo in reducing Interleukin-8 (IL-8) and C-reactive protein (CRP). Normalization in CRP occurred in 90%, 52%, and 19% of patients, respectively. Both NAC doses improved expectoration, cough, and FEV_1_ (56). In another trial (15 patients per group), NAC 1,200 mg and 600 mg significantly reduced IL-8 and malondialdehyde (*p* < 0.001), improved partial pressure of oxygen in arterial blood (PaO_2_) (*p* < 0.001), and increased FEV_1_, FVC, and FEV_1_/FVC versus placebo (57).

In hospitalized patients, 600 mg twice daily NAC (*N* = 21) added to standard care significantly improved PaO_2_, partial pressure of carbon dioxide (PaCO_2_), Oxygen (O_2_) saturation, wheeze, and dyspnea by day 3, with further gains by day 7 as compared to standard care (*n* = 19) (58).

N-acetylcysteine also enhanced antibiotic bioavailability and bacterial clearance. EPAC (erythromycin propionate–N-acetylcysteinate) achieved faster bacterial clearance and better symptom resolution (fever, sputum viscosity, mucus clearance) than erythromycin stearate (59), while NAC 600 mg BID accelerated bacterial clearance in acute bronchitis versus standard of care (70% vs. 36%; *p* < 0.03) (60).

In acute-on-chronic bronchitis, NAC 600 mg/day improved symptoms, sputum, and PEFR versus placebo (61), though Black et al. found no improvement in FEV_1_, arterial oxygen saturation (SaO_2_), or hospital stay with NAC 600 mg BID in AECOPD (62). Zambon Pharmaceuticals reported that NAC 600 mg/day, but not 1,200 mg/day, improved FEV_1_, FEV_6_, and FEV_1_/FVC versus placebo, though breathlessness cough sputum score (BCSS) scores were unchanged (63). Another double-blind RCT (42 COPD patients) showed non-significant but greater improvement in FEV_1_ (366.3 vs. 201.8 ml, *p* = 0.076) and blood gasses with NAC 600 mg/day versus placebo (64).

### 5.2 Summary of meta-analyses and reviews

A meta-analysis of 15 RCTs (*N* = 905) found NAC 600 mg/day significantly improved symptoms, FEV_1_, FEV_1_/FVC, GSH-ST activity, and reduced hydroxyl and superoxide anion free radicals versus controls (65).

Papadopoulou et al. (66) have published a meta-analysis of 2,192 participants from 24 studies (14- Double blinded, five single-blinded, five blinding unclear) reviewed six mucolytics - NAC (*n* = 10), Ambroxol (*n* = 5), Erdosteine (*n* = 5), Bromhexine (*n* = 2), and others (*n* = 2). The review concludes that mucolytics:

1. Increased treatment success (relative risk 1.37, 95% CI 1.08–1.73, *n* = 383).
2. Improved overall symptom scores (standardized mean difference 0.86, 95% CI 0.63–1.09, *n* = 316).
3. Reduced cough at follow-up (relative risk 1.93, 95% CI 1.15–3.23).
4. Improved ease of expectoration (relative risk 2.94, 95% CI 1.68–5.12).

### 5.3 Consensus of the expert panel

**FIGURE 3:**
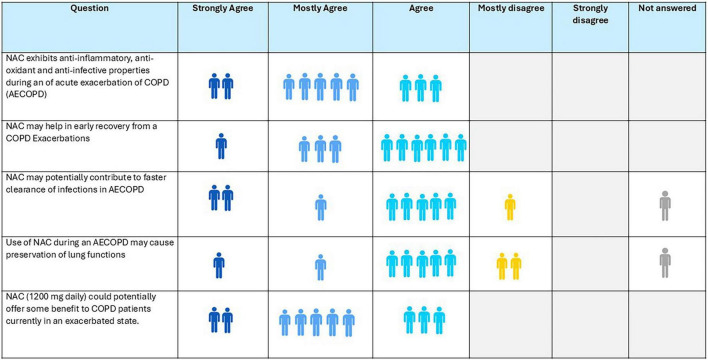
Opinion of experts about role of N-acetylcysteine (NAC) during an ongoing acute exacerbations of chronic obstructive pulmonary disease (AECOPD).

The expert panel reached a strong consensus that the pleiotropic properties of oral NAC contribute to early recovery of AECOPD and 80% agreed that it may facilitate clearing the infection faster (Figure 3). Around 70% concurred that it demonstrates a trend toward preservation of lung function and all agreed that 600 mg/day NAC may confer greater benefit to patients during an AECOPD than 1,200 mg/day.

**Recommendation:**

1. N-acetylcysteine is useful for symptom management and early recovery during AECOPD, leveraging its anti-inflammatory, antioxidant, anti-infective, and mucolytic properties.
2. Oral NAC at a dose of 600 mg/day is recommended for patients experiencing an acute exacerbation.
3. NAC may potentially aid faster infection clearance.
4. Evidence for lung function preservation exists as a non-statistically significant numerical trend. More studies with larger sample sizes are needed before definitive conclusions can be made.
5. Laboratory-based outcomes show reductions in inflammatory mediators, which may translate to clinical benefit, but these findings should be interpreted cautiously.
6. NAC should be used adjunctively along with bronchodilators, corticosteroids, and other guideline-recommended interventions.

## 6 Role of NAC in tuberculosis

Mycobacterium tuberculosis (MTB) remains a major treatment challenge globally due to high microbial resistance, lung inflammation and hepatotoxicity of anti-tubercular treatment (ATT). NAC, with its pleotropic properties, has been explored for multifaceted roles including early bacterial clearance, protection of lung tissue from damage, and mitigation of anti-tubercular drug-induced hepatotoxicity. It is important to present *in vitro* and animal studies here as there is limited evidence in human clinical trials.

### 6.1 *In vitro* and animal studies

#### 6.1.1 Antimicrobial activity of NAC

N-acetylcysteine significantly reduces metabolic activity of MTB and has direct anti-microbicidal action. In a murine model, NAC-treated mice exhibited significantly lower bacterial lung load by almost 90% (Lung CFU counts were reduced by a biologically significant > 1 log_10_) compared to untreated controls (67). An *in vitro* study on blood cells of patients with Type 2 Diabetes Mellitus (DM) and healthy volunteers showed that NAC has anti-TB microbial properties on its own and significantly inhibited the growth of MTB as compared to sham treated media by about 50% at 8 days and 70% at 15 days, respectively. In Type 2 diabetes (T2DM) patients, NAC alone caused a 4-fold reduction in *M. tuberculosis* survival (68).

### 6.2 Synergistic role of NAC with ATT

Khameneh et al. demonstrated that both NAC and Vitamin C showed synergistic effects on the anti-bacterial properties of ATT with Vitamin C being much more efficient than NAC (69). NAC acts synergistically with all the three AKT drugs i.e., Isoniazid (INH), Rifampicin (RIF), and Ethambutol (ETB) and enhanced bacterial clearance by 10-fold and 12-fold, respectively as compared to sham treated media (68). Despite severe infection and relatively weak granulomas formation, these effects of NAC were also seen in cells from patients with Type 2 DM (68). In another study the INH/RIF/NAC combination achieved the benchmark of killing 10^6^ MTB 10 days sooner (at 11 days) than the INH/RIF combination (at 21 days) (70). The synergy was also evident in drug-resistant MTB strains. The multidrug treatment regimens of OKE (ofloxacin/kanamycin/ethionamide) and MAC (moxifloxacin/amikacin/clofazimine) against drug-susceptible, MDR, and XDR TB were also studied. The addition of NAC significantly accelerated the killing benchmark by 10 days for OKE and 5 days for MAC against drug-susceptible and MDR strains, with the exception of the MAC combination against the XDR strain. By increasing the respiration of MTB NAC also keeps them active and prevents them from developing resistance (70).

### 6.3 Effect of the antioxidant property of NAC in TB

The TB plasma showed increased oxidative stress (Lipid Peroxidation, ROS) and reduced total antioxidant status. NAC significantly reduced the accumulation of ROS, lipid peroxidation and DNA oxidation by approximately 87%, 79%, and 77%, respectively, reducing oxidative stress and increasing antioxidants (67).

Controlled study of induced granulomas treated with NAC showed 2-fold increase in GSH, 3-fold increase in Interferon-γ, 2- and 3-fold reduction in tumor necrosis factor-α (TNF-α) at 8 and 15 days, reduced reactive oxygen ions and reduced necrosis as compared to controls (68).

### 6.4 Summary of RCTs

In the RIPENACTB trial, adjunct NAC treatment with 1,200 mg twice a day in hospitalized HIV/TB patients significantly reduced circulating lipid peroxidation (malondialdehyde (MDA) levels by approximately 40%–50%) and increased antioxidant defenses, including glutathione (GSH, by approximately 30%–40%), compared to baseline and the standard care group. NAC also significantly reduced the concentration of IL-1RA which is a marker of oxidative stress and increased the concentration of plasma protective cytokines IL-17A (*p* = 0.012) and Vascular Endothelial Growth Factor (*p* = 0.043) (71).

Mahakalkar et al. from India conducted an RCT and showed that 600 mg two tablets daily NAC supplementation alongside standard TB treatment led to faster sputum conversion (95.8% in NAC group vs. 58.3% in control group at 3 weeks) and earlier radiological improvement by significantly reducing the cavity surface area and increasing glutathione peroxidase levels in the NAC group (72). The RIPENACTB trial reported that 1,200 mg BID NAC was safe in HIV-positive TB patients, though it did not show a significant impact on sputum conversion rate (73).

A Phase 2 RCT amongst 140 adults highlighted potential role of high dose NAC (1,200 mg BID) in preserving lung functions in adults with moderate to far-advanced pulmonary tuberculosis. NAC significantly boosted total glutathione (*p* < 0.0001) and suppressed pro-inflammatory TNF-α (*p* = 0.011) and accelerated hemoglobin recovery in patients with advanced pulmonary TB but did not improve sputum culture conversion or recurrence rates. However, NAC was associated with significantly greater improvement in lung function (FEV_1_% and FVC%), particularly in those with severe baseline impairment, with effects persisting beyond treatment. Safety outcomes were similar between groups. This study indicates NAC’s potential role in preventing post-TB Lung sequelae which needs to be validated through further large-scale studies (74, 75).

In a recent quasi-experimental study in newly diagnosed adults TB patients, the IL-2 levels observed over 2 weeks after NAC administration showed that the median IL-2 levels were higher in the treatment group than in the control group. IL-2 plays a significant role in the immune response against MTB by enhancing lymphocyte proliferation and by autocrine and paracrine effect. Significant increase in IL2 can potentially help in the control of MTB infection (76).

### 6.5 Role of NAC and anti-tuberculosis drug induced liver injury (AT-DILI)

#### 6.5.1 In vivo

Animal studies have demonstrated the hepatoprotective effect of NAC by blocking lipid peroxidation and reducing superoxide dismutase activities induced by INH and Rifampicin. Liver of the mice exposed to INH + Rifampicin without receiving NAC showed significant inflammation and hepatic necrosis (77, 78).

#### 6.5.2 Summary of RCTs

Three independent prospective placebo controlled RCTs with a cumulative participant population of 90 in the NAC group and 93 in the control group have reported that the NAC groups showed remarkably lower level of liver enzymes at 2, 4, and 8 weeks as compared to groups that did not receive NAC (79–81). Amongst the controls, 12 and 14.3% developed hepatotoxicity as against none in the NAC group (79, 80). In 2020, Fox et al. reported a case of severe hepatoxicity due to AKT in a 30-year-old woman with multiple morbidities whose Acute Drug Induced Liver Injury responded well to intravenous NAC (82).

Moosa et al. (83) found that administration of intravenous NAC shortened the hospital stay of TB patients with AT-DILI (9 days; NAC group and 18 days; placebo arm). The Hazard Ratio (HR) for hospital discharge was 1.73 (95% CI, 1.13–2.65). There was no difference in time to normalization of liver enzymes which Hampannavar et al. (84) asserts could be because of the antiretroviral drugs that the 87% HIV-positive participants were probably on.

### 6.6 Summary of meta-analyses and reviews

A literature review by Yudhawati et al. summarized the positive role of NAC in TB due to its immunomodulatory, anti-inflammatory and antimicrobial properties (85). Ejigu et al., Mapamba et al. have concluded that NAC is a safe drug, helps protect cells from damage caused by oxidative stress, reduces inflammation, increases GSH levels, has direct antibacterial effects, and also regulates the immune system, helping it function effectively. NAC enhances the effectiveness of anti-TB drugs and protects the liver from damage caused by these medications. However more clinical trials are required to consolidate the role of NAC as an adjunct to ATT in management of TB (86, 87).

In summary,

1. Multifaceted activity: NAC provides immunomodulatory, anti-inflammatory, and antimicrobial benefits.
2. Oxidative protection: It helps protect cells from damage caused by oxidative stress by increasing Glutathione (GSH) levels.
3. Direct antibacterial effect: Research suggests NAC possesses direct properties that inhibit bacterial growth though this is less consistent across all studies.
4. Synergy with ATT: NAC enhances the overall effectiveness of standard anti-TB drugs.
5. Hepatoprotection: It serves a protective role for the liver, mitigating damage often caused by the toxicity of anti-TB medications.
6. Safety profile: Current conclusions indicate that NAC is a safe drug for use in this context.

### 6.7 Consensus of the expert panel

**FIGURE 4:**
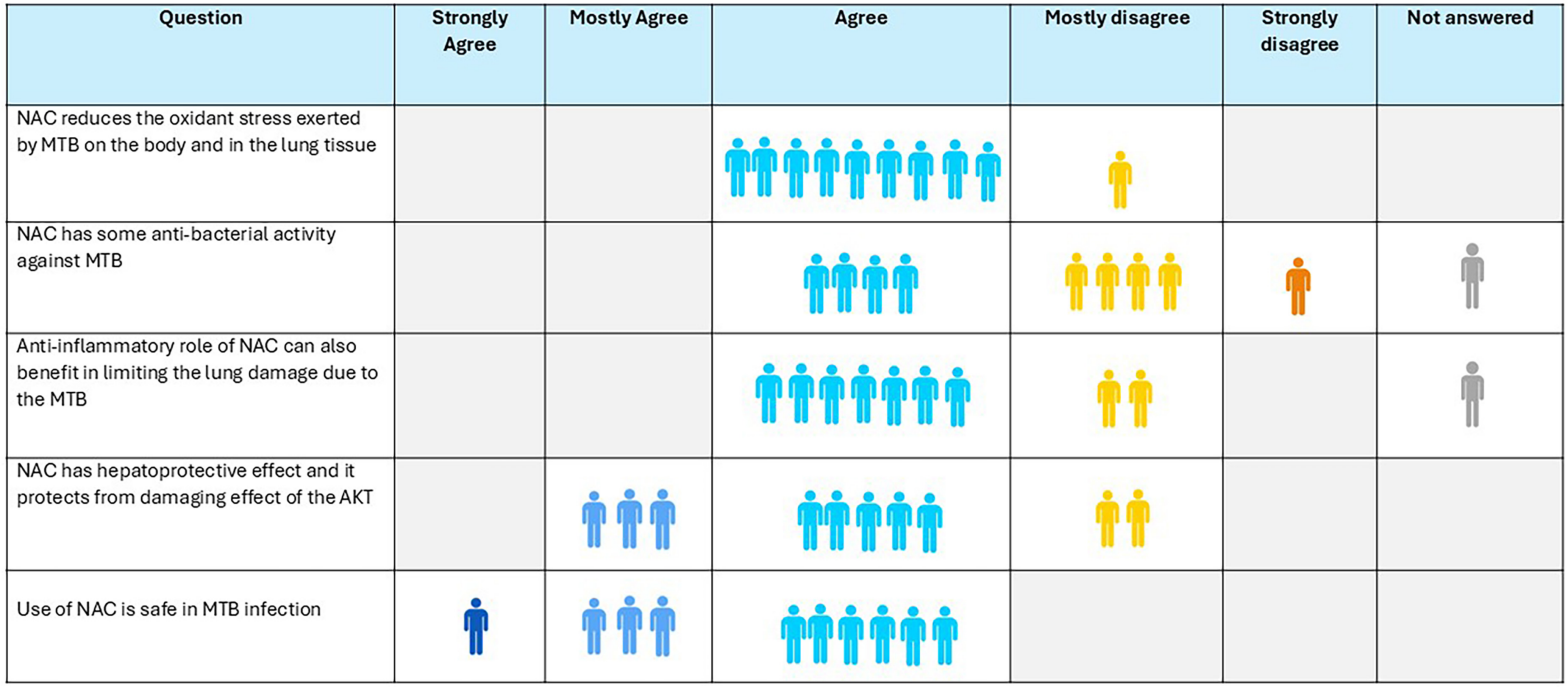
Opinion of the experts on role of N-acetylcysteine (NAC) in tuberculosis.

Ninety percent of panel experts agreed that NAC reduces oxidative stress in the lungs, and 70% concur that it may help limit lung damage caused by MTB (Figure 4). NAC has a hepatoprotective role in AT-DILI was agreed upon by 80% of the experts, while only 40% endorsed its potential inherent anti-mycobacterial properties. All experts supported the safety of NAC in treatment of MTB infection.

**Recommendation:**

1. N-acetylcysteine is safe in TB patients, may reduce oxidative stress, and may limit lung tissue damage and protect against drug-induced liver injury.
2. Its inherent antimicrobial activity against MTB needs to be investigated further.
3. NAC has the potential to prevent AT-DILI.
4. Given the limited and small-scale clinical evidence, the expert group did not provide any formal dosing recommendations.
5. The expert panel recommends robust research to generate evidence for clinical efficacy, optimal dose, and duration for the use of NAC alongside standard anti-TB therapy.

## 7 Role of NAC in non- cystic fibrosis bronchiectasis

Though the exact prevalence of bronchiectasis in the general population in India is unknown, the EMBARK study has highlighted the fact that the population in India that suffers with bronchiectasis is a younger population [Average age 57 vs. 67 years (*p* < 0.05) in Europe] with a more severe form of the disease and differs from the European etiotypes and phenotypes (88).

International guidelines acknowledge role of mucoactives like inhaled mannitol, recombinant DNase, hypertonic saline, and nebulized hypertonic saline in the management of non-cystic fibrosis (NCF) bronchiectasis (89, 90). NAC being a potent mucoactive agent may have a beneficial role.

### 7.1 Summary of RCTs

Role of NAC in NCF bronchiectasis has recently been explored by two RCTs. The BENE study, a multicentric, prospective, randomized, controlled trial with 600 mg BD (*N* = 161, NAC-81, control-80) recruited patients who had at least two exacerbations in the previous year and were stable for at least 4 weeks before their recruitment, showed significant reduction in number of exacerbations in the NAC group (1.31 vs. 1.98 year in control; *p* = 0.001), higher number of exacerbation free patients (24.7% in NAC Vs. 11.3% in control; *p* = 0.02), delayed first exacerbation and decreased volume of sputum over 12 months.(91)

Jayaram et al. in a small placebo-controlled study with sample size NAC (*n* = 9) vs. placebo (*n* = 8), have shown that neutrophil elastase, a surrogate marker of exacerbation, decreased by 47% in the 2,400 mg/day NAC group as compared to placebo over 6 weeks (*p* = 0.045). The NAC group also showed significant improvement in median FEV_1_ and FVC (+80 and +50 mL, respectively) at 6 weeks, compared with a decline in the placebo group (FEV_1_−100 mL and FVC −200 mL).(92)

In a retrospective multi-center study of 2,461 bronchiectasis patients over 2 years, Oscullo et al. compared the efficacy of 1,200 mg/day (*n* = 116) versus 600 mg/day of NAC (*n* = 252).

Propensity score matching (*n* = 104 vs. *n* = 219) revealed that the 1,200 mg/day dose significantly reduced:

1. Total exacerbation rates: −54.1% (*P* = 0.002)
2. Exacerbation frequency: −48.6% (*P* = 0.01)
3. Hospitalizations: −29.9% (*P* = 0.038)

The higher dose also improved sputum characteristics compared to the 600 mg/day group, with a 24.3% decrease in high-volume sputum (>20 cc/day; *P* = 0.001) and an 8.5% reduction in mucopurulent sputum (*P* = 0.041) (93).

ERS Clinical Practice Guidelines (2025): The European Respiratory Society updated its management guidelines, issuing a conditional recommendation for the use of mucoactive drugs like NAC in specific NCFB phenotypes, particularly those with high sputum production and frequent exacerbations (94).

### 7.2 Consensus of the expert panel

**FIGURE 5:**
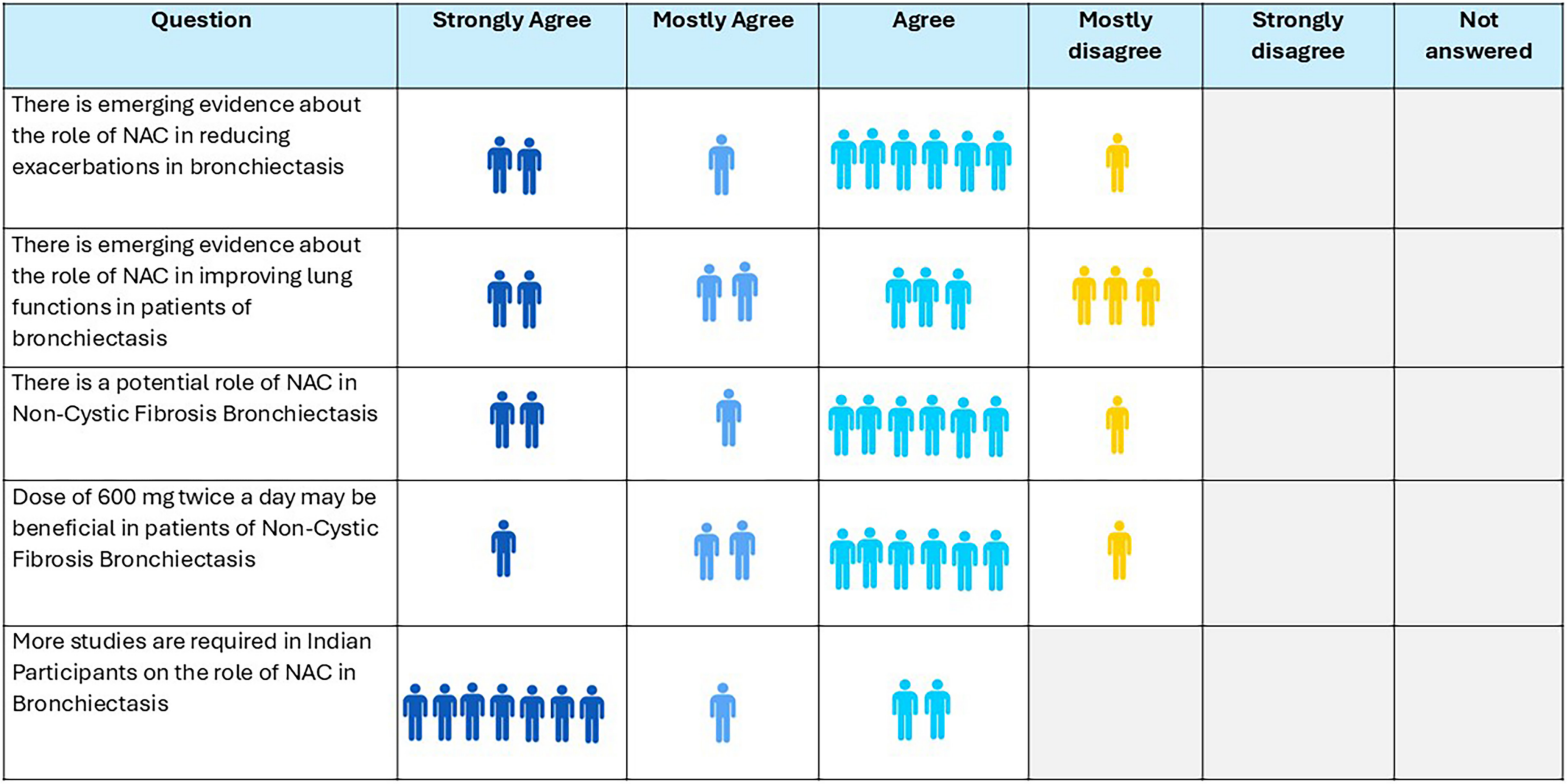
Opinion of the experts on role of N-acetylcysteine (NAC) in non-cystic fibrosis bronchiectasis.

Ninety percent of experts came to a consensus that 600 mg of NAC, taken twice daily, may be beneficial for patients with NCF bronchiectasis, and emerging evidence on its role in reducing exacerbation supports the opinion of 70% experts that NAC improves lung functions in these patients; however, experts expressed the opinion that more studies are required (Figure 5).

**Recommendation:**

1. NAC could be considered for patients with NCF bronchiectasis who experience ≥ 2 exacerbations per year.
2. A dose of 600 mg twice daily is recommended.
3. Evidence remains limited and more robust studies are required.

## 8 Role of NAC in cystic fibrosis

Cystic Fibrosis (CF) is an autosomal recessive condition caused by Cystic Fibrosis Transmembrane Conductance Regulator (CFTR) gene mutation. This mutation leads to thick, stagnant mucus, promoting bacterial biofilms. Abnormal neutrophil recruitment in CF airways increases oxidative stress, causing redox imbalance and elastase/IL-8 release. This results in tissue damage, high sputum viscosity, and impaired airway clearance.

While extrapolated data from UK/US, CF clinics suggest a prevalence of 1:12,000–1:40,000 among Indian children (95), a 2020 North Indian study revealed a high CFTR mutation carrier rate of 1:22, signaling a potentially higher prevalence (96).

Adding to the disease burden, poor survival is attributed to delayed diagnosis, malnutrition, bacterial colonization, and related complications (95). Medhi et al. reported that at diagnosis (mean age 19 years; range 16–27), patients were already sputum-positive for *P. aeruginosa* (68.7%), *Methicilline Sensitive Staphylococcus Aureus* (MSSA) (31.2%), and *Methicilline Resistant Staphylococcus Aureus* (MRSA) (12.5%) (97).

N-acetylcysteine (NAC) benefits cystic fibrosis by mitigating oxidative stress and inflammation through antioxidant, anti-inflammatory, and anti-LPS mechanisms, partly via modulation of neurokinin A and IL-6. It also exhibits anti-biofilm activity, reducing bacterial resistance and enhancing antibiotic efficacy against *P. aeruginosa, S. epidermidis*, and *Acinetobacter*, both alone and in combination with NSAIDs (98).

### 8.1 *In vitro* studies

*In vitro* studies show that *P. aeruginosa* and other bacterial pathogens are present in nearly all CF patients, with widespread biofilm formation (99). NAC disrupts biofilms by penetrating and killing embedded bacteria, detaching mature biofilms, inhibiting bacterial adhesion, and reducing extracellular polymeric substance (EPS) production (100, 101).

### 8.2 Summary of RCTs

Early studies using low-dose NAC (200 mg TID) showed no significant benefit over placebo in PEFR, weight, antibiotic use, cough, sputum viscosity, or symptom perception (102). Ratjen et al. later found that Ambroxol and NAC (200 mg TID) prevented FEV_1_ decline over 12 weeks, unlike placebo (103).

Two randomized trials reported significant improvements in FEV_1_ and FVC with NAC (200 mg TID or 400 mg BID) in CF patients, with or without *P. aeruginosa*, particularly during infection-prone autumn months (104, 105).

Tirouvanziam et al. showed that NAC (600–1,000 mg/day) restored antioxidant balance and reduced inflammation, though without lung function improvement (106).

A phase II trial using high-dose NAC (2,800 mg/day, 12 weeks) increased sputum glutathione but did not affect inflammatory markers or lung function (107). In contrast, NAC 900 mg TID reduced FEV_1_ decline (by 150 mL) versus placebo over 24 weeks, though primary endpoints were unmet due to underpowering (108).

Cystic fibrosis patients with chronic *P. aeruginosa* receiving NAC (2,400 mg/day, 4 weeks) showed improved serum antioxidants, reduced oxidative stress, and better FEV_1_% than controls (109).

### 8.3 Summary of meta-analyses and reviews

In an extensive review focused on role of NAC in pediatric respiratory conditions Beneditti et al. summarize that (110)

1. N-acetylcysteine is a useful adjunct for improved health outcomes in CF.
2. The dosage may be dose base at 10–30 mg/kg but for older children 200 mg TID would be an appropriate dose.

### 8.4 Consensus of the expert panel

**FIGURE 6:**
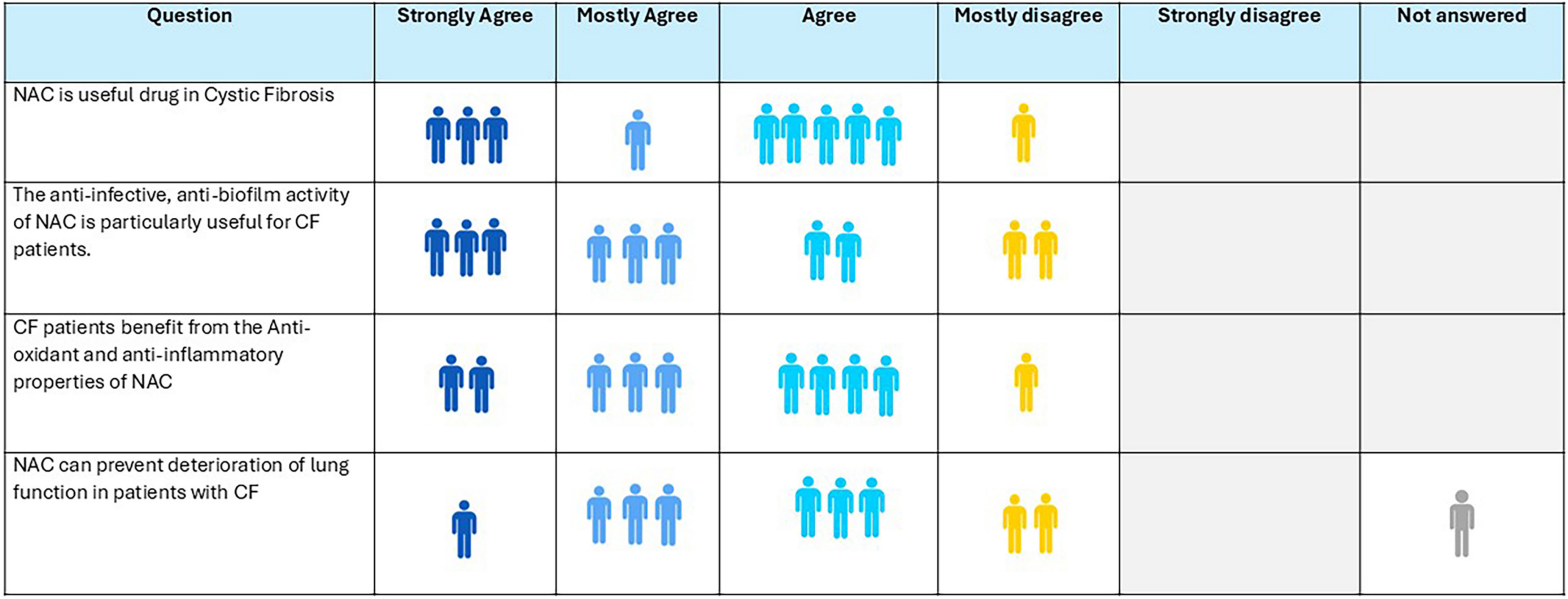
Opinion of the experts on role of N-acetylcysteine (NAC) in cystic fibrosis.

About 90% of experts expressed that NAC could be beneficial in cystic fibrosis, primarily due to its antioxidant and anti-inflammatory effects (Figure 6). The experts emphasized NAC’s potential to prevent and disrupt biofilms-an aspect acknowledged by 80% of respondents as contributing to its benefit in CF. Additionally, 70% agreed that NAC may help slow the decline in lung function associated with the condition.

**Recommendation:**

1. In CF, evidence supports both symptomatic and disease-modifying benefits, particularly in preventing biofilm formation and preserving lung function.
2. NAC in a weight-based dosage for younger children and age-appropriate dose for older patients (600–1,200 mg per day) is recommended to be given to patients of CF.

## 9 Role of NAC in bacterial and viral respiratory infections

Bacterial biofilms are complex structures formed by microorganisms that adhere to a surface and are encased in a self-produced extracellular matrix (111). This matrix, primarily composed of EPS, provides a protective environment for the bacteria within, making them resistance to antimicrobial agents and are present in both acute and chronic infections of community acquired pneumonia (CAP), CF and COPD (99).

### 9.1 Role of NAC in inhibiting and disrupting biofilm

*In vitro*, NAC inhibits biofilm formation and disrupts existing biofilms of a variety of bacteria, including *P. aeruginosa* and *S. aureus* both MRSA and MSSA (100, 112–116). It reduced antibiotic resistance and directly reduces the viability of certain bacteria (101, 113, 115). These studies suggest usefulness of NAC in managing chronic respiratory diseases like CF, bronchiectasis and COPD, often complicated by bacterial infections.

### 9.2 Antioxidant property of NAC and its role in management of pneumonias

Duflo et al. have demonstrated increased oxidative stress in the alveoli and plasma in ventilator-associated pneumonia (VAP) (116). In community acquired pneumonia (CAP), the increased oxidative stress defines the severity of the disease (117, 118). In a RCT amongst patients hospitalized for CAP, plasma levels of malondialdehyde (MDA) and TNF-α decreased more (*P* < 0.05 MDA:p 0.004, TNF-α: *p* < 0.001) in the NAC group (*n* = 21) than the non-NAC group (*n* = 18), and there was a reliable increase in total antioxidant capacity (TAOC) content (p 0.005). Though the NAC group did not show a greater improvement on computed tomography scores, addition of NAC to the management protocol of CAP patients may help limit oxidative and inflammatory damage in pneumonia patients (119). In a placebo-controlled RCT, Sharafkhan et al. (120) reported that mechanically ventilated patients receiving NAC had significantly lesser incidence of VAP (26.6% vs. 46.6%; *p* = 0.032), shorter stay in the ICU (14.36 ± 4.69 days vs. 17.81 ± 6.37 days, *p* = 0.028), shorter hospital stay (19.23 ± 5.54 days vs. 24.61 ± 6.81 days; *p* = 0.03), longer time to VAP (9.42 ± 1.9 days vs. 6.46 ± 2.53 days; *p* = 0.002) and better complete recovery in NAC group (56.6% vs. 30%; *p* = 0.006) compared with placebo.

### 9.3 Role of NAC in ENT infections

Chronic infections and bacterial antibiofilm development are therapeutic challenges in chronic rhinosinusitis, otitis media with effusion, or cholesteatoma. NAC helps with mucolysis and improving mucociliary clearance.

Corticosteroid and NAC nasal douching significantly reduced symptom severity more than two-fold, resolved mucosal edema, and restored mucociliary function in recurrent acute rhinosinusitis patients compared to steroid with ambroxol, leading to better long-term satisfaction (121). Children with otitis media showed better recovery rate and lesser need for surgery in the oral NAC group as compared to controls (122, 123). Reduced saccharine test time (STT) in the NAC group indicates that NAC, not placebo, improves mucociliary clearance activity (124). NAC acts synergistically with levofloxacin causing early and better resolution of chronic sinusitis (125).

A detailed review of literature has found increased oxidative stress in acute as well as chronic infections of the ear, nose, and sinuses and the rationale for adding an antioxidant to the therapy (126).

### 9.4 Role of NAC in viral infections including COVID 19

N-acetylcysteine inhibits replication of influenza A and other respiratory viruses in cell cultures and suppresses inflammatory mediators (CXCL8, CXCL10, CCL5, H_2_O_2_, IL-6) by blocking NF-κB and p38 MAPK activation (127, 128). Animal studies show NAC protects against pulmonary inflammation, edema, MPO activity, cellular infiltration, and cytokine elevation (TNF-α, IL-6, IL-1β, CXCL10) in swine influenza–induced lung injury (129). A community-based RCT by Flora et al. demonstrated reduced incidence of influenza-like illness in elderly individuals receiving NAC (130).

N-acetylcysteine inhibits multiple Influenza A strains, and intravenous NAC improves outcomes in acute respiratory distress syndrome and acute lung injury (131, 132). It is safe and may be beneficial in COVID-19 due to its anti-inflammatory, immunomodulatory, and antiviral properties that mitigate the SARS-CoV-2–induced cytokine storm (133). A meta-analysis of 14 studies (*n* = 20,980) showed NAC significantly reduced CRP and D-dimer levels, increased P/F ratios, and decreased hospital stay and mortality in COVID-19 patients (134).

N-acetylcysteine has a high safety profile, and the studies do not report any major or serious adverse effect of NAC.

Overall, NAC shows promise as a potential safe and effective treatment for both bacterial and viral infections. However, more clinical research is needed to determine its definitive role in managing these infections.

### 9.5 Consensus of expert panel

**FIGURE 7:**
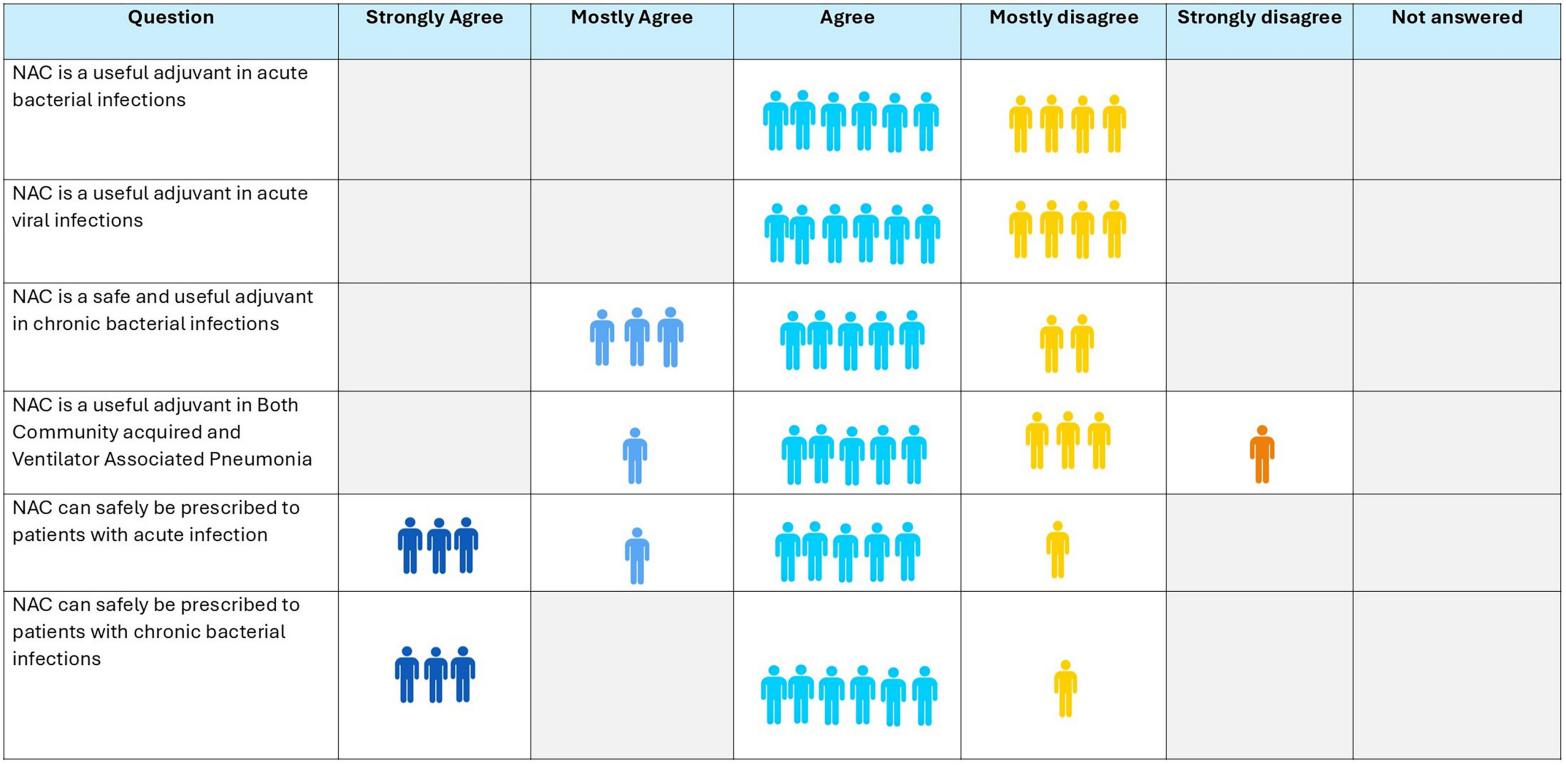
Opinion of the experts on role of N-acetylcysteine (NAC) in bacterial and viral infections.

Eighty percent of experts concurred that NAC can be a useful adjunct in the treatment of chronic bacterial infections and 90% experts agreed that NAC is a safe drug to be administered in acute and chronic bacterial infections (Figure 7). Around 60% reached a consensus that it could be a useful adjuvant in acute bacterial and viral infections and 60% agreed that it has a role in CAP but not in VAP.

**Recommendation:**

1. NAC is a safe drug and may be used as adjuvant therapy in acute and chronic infections.
2. Larger studies are required to substantiate its role in acute and chronic bacterial and viral infections.

## 10 Role of NAC in interstitial lung diseases

In India, ILD incidence is 10.1–20.2 and prevalence 49.0–98.1 per 100,000, accounting for 0.45–0.89 million cases nationally (135). NAC has been studied extensively to see if it can benefit patients with ILD.

### 10.1 Summary of RCTs

Multiple RCTs have evaluated NAC in interstitial lung diseases, particularly idiopathic pulmonary fibrosis (IPF). In the IFIGENIA study, adding NAC 600 mg/day to prednisone and azathioprine slowed lung function decline and improved Diffusing Capacity of the Lung for Carbon Monoxide (DLCO), especially in early IPF (136, 137).

The PANTHER trial (IPFnet) compared NAC, combination therapy (prednisone + azathioprine + NAC), and placebo in 236 patients (138). The combination arm was terminated early due to severe adverse events and deaths, while NAC alone showed no significant effect on lung function, though secondary endpoints (6MWD, QoL) improved modestly (139). *Post hoc* analysis revealed genotype-dependent response, with TOLLIP-TT carriers (∼25%) benefiting from NAC, no effect in TOLLIP-CT, and harm in TOLLIP-CC genotypes (140). Another retrospective study found better survival in UIP-type IPF with positive antinuclear antibody, possibly due to higher TOLLIP-TT prevalence (141).

An RCT combining NAC with pirfenidone found no additional lung function benefit but increased photosensitivity (142). Conversely, a retrospective analysis reported fewer adverse reactions, improved QoL, and longer survival with NAC 600 mg TID + pirfenidone versus pirfenidone + budesonide (143).

### 10.2 Summary of meta-analyses and reviews

Systematic reviews and meta-analyses suggest that

1. N-acetylcysteine modestly attenuates lung function decline (FVC, DLCO).
2. Preserves endurance (6MWT).
3. Slows IPF progression as indicated by stable PaO_2_ (144, 145).
4. Some studies reported increased hospitalization rates (146).

A review of five studies found no additional benefit of combining NAC with pirfenidone in slowing lung function decline (147).

### 10.3 Sarcoidosis-associated ILD summary of randomized controlled trials

Hamzeh et al. could not demonstrate any benefit of NAC treatment, compared with placebo, on lung function, quality of life, or inflammatory markers in patients with sarcoidosis-associated ILD (148).

### 10.4 Consensus of expert panel

**FIGURE 8:**
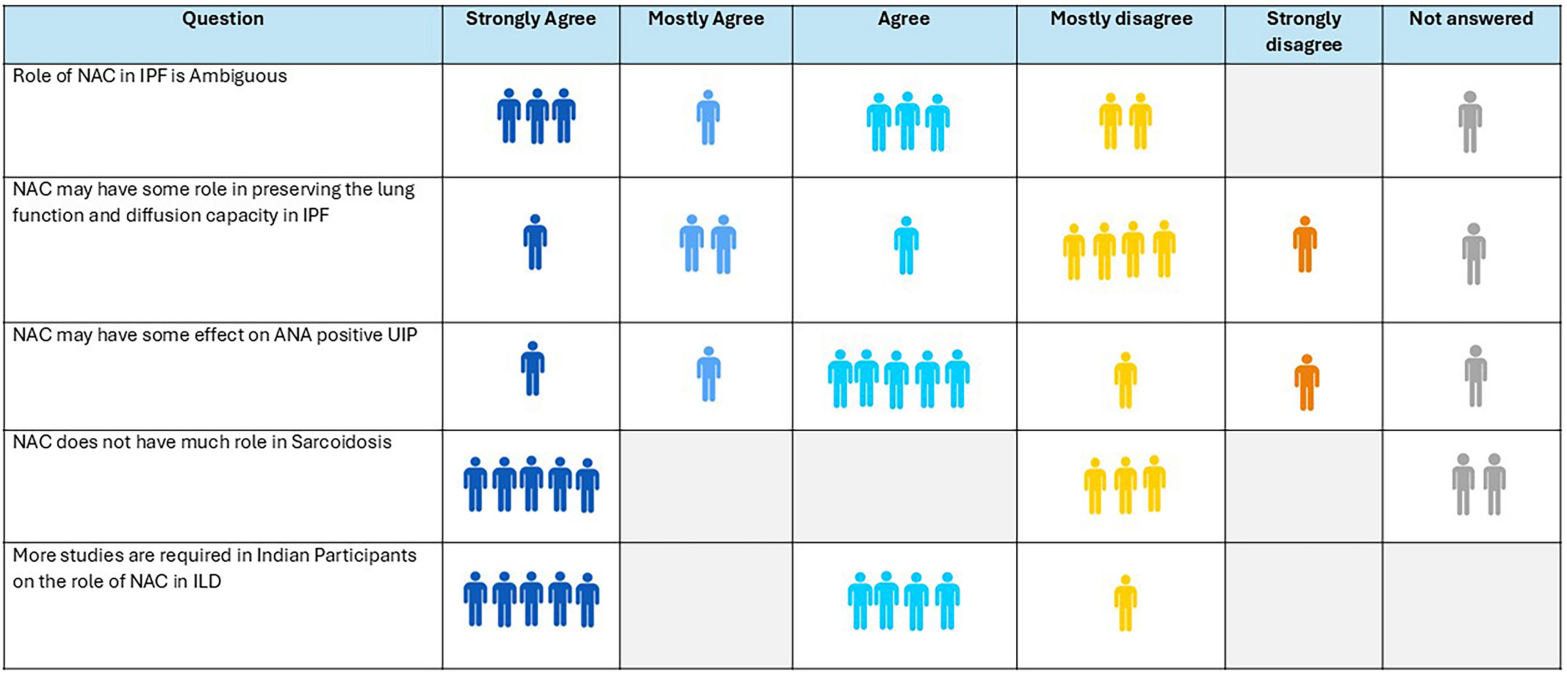
Opinion of the experts on role of N-acetylcysteine (NAC) in interstitial lung disease (ILD).

Seventy-eight percent of respondents supported that the role of NAC in ILDs remains unclear, and 40% felt it may help preserve lung function in ILD (Figure 8). Seventy percent agreed it may have some effect in antinuclear antibody-positive usual interstitial pneumonia (UIP), while 5 of the 8 respondents agreed it has no role in sarcoidosis. Ninety percent agreed that further studies are needed to assess its role in the Indian ILD population.

**Recommendation:**

Based on the current evidence and expert opinions, NAC cannot be recommended as a standard therapy in ILDs.

## 11 NAC in asthma

Asthma prevalence varies widely across countries and regions due to geographical and socioeconomic factors. The Global Asthma Network estimates a 3.3% prevalence among Indian adults (149). GBD 2021 data report 32.01 million asthmatics in India, accounting for 12.3% of global cases and 46.08% of global asthma deaths, the highest worldwide. While NAC’s benefits are well established in COPD, evidence supporting its use in asthma remains limited.

### 11.1 Animal studies

Animal and *in vivo* studies have shown enhanced steroid responsiveness, lower airway hyperresponsiveness (AHR), and reduction in inflammatory cells and oxidative stress in asthma (150–153). However, improvement in clinical outcomes of asthma exacerbations was not observed with 600 mg twice-daily oral NAC, according to a single-blinded RCT by Aliyali et al. (154).

### 11.2 Summary of RCTs

In a single double-blind crossover RCT by Carlsten et al. with 26 participants showed that using NAC as an antioxidant (600 mg TID for 6 days) significantly decreased hyperresponsiveness by 20% in patients exposed to diesel exhaust particles compared to placebo. Participants also reported a mean 58% reduction in the use of SABA (155).

However, a few case reports have advised caution in asthma patients receiving NAC for paracetamol poisoning, as severe bronchoconstriction was observed following IV infusion (156).

### 11.3 Consensus of expert panel

**FIGURE 9:**
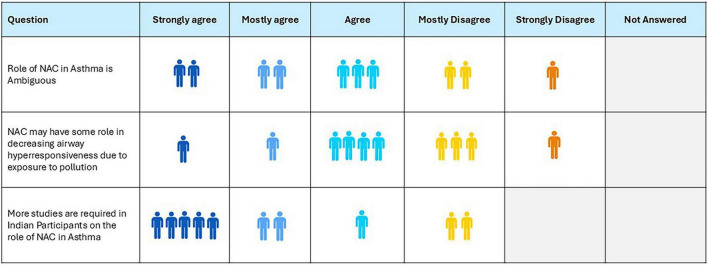
Opinion of the experts on role of N-acetylcysteine in asthma.

Seventy percent of experts agreed that the role of NAC in asthma is ambiguous, though 60% felt it may reduce air pollution–induced hyperresponsiveness. Eighty percent agreed that further studies are needed (Figure 9).

**Recommendation:**

The role of NAC in asthma management is underexplored, and at present, it is not recommended for use in asthma.

## 12 Overall safety of NAC in respiratory conditions

Calverly et al. (157) have reviewed forty one articles including major RCTs that have studied the effect on NAC on respiratory conditions including chronic obstructive pulmonary disease, idiopathic pulmonary fibrosis, bronchiectasis, chronic bronchitis and cystic fibrosis with respect to the side effects of NAC at doses between 600 mg per day to 3,000 mg per day. Overall, whether used for acute exacerbations or over extended periods at higher doses (e.g., 600–1,200 mg/day for COPD), NAC is generally very well-tolerated. In major clinical trials, the overall incidence of adverse events for patients taking oral NAC has consistently been comparable to those taking a placebo. Even in patients with tuberculosis, NAC at doses of 600/1,200 or 1,800 mg per day are found to be generally safe (85).

### 12.1 Key safety observations

1. Gastrointestinal effects: The most commonly reported side effects are mild and gastrointestinal in nature. These can include nausea, vomiting, diarrhea, and mild abdominal discomfort or dyspepsia.
2. Dermatological effects: Less frequently, patients may experience mild skin reactions, such as a temporary rash or pruritus (itching).
3. Respiratory reactivity: When used via nebulization, inhaled NAC carries a rare risk of inducing bronchospasm. This requires caution, particularly in patients with hyperreactive airways, such as those with asthma.

In summary, current clinical data confirms that NAC is a safe adjunctive therapy with a minimal risk of severe adverse effects for chronic respiratory conditions.

## 13 Summary

N-acetylcysteine (NAC) is a potent, yet underutilized, therapeutic agent extending far beyond its mucolytic effects. Its antioxidant, anti-inflammatory, cytoprotective, immunomodulatory, antibacterial, and antibiofilm properties make it a valuable adjuvant in diverse chronic and infectious respiratory diseases.

N-acetylcysteine benefits patients with stable COPD, exacerbations, and tuberculosis by mitigating *M. tuberculosis*–induced injury and reducing hepatotoxicity from anti-TB drugs. In cystic fibrosis, NAC provides anti-inflammatory, antioxidant, and antibiofilm effects, with emerging evidence supporting its role in non-CF bronchiectasis. For chronic refractory upper airway infections, such as sinusitis and otitis media, NAC enhances antibiotic efficacy, disrupts biofilms, and improves mucociliary clearance through increased ciliary activity.

Given its excellent safety profile, NAC should be considered an effective adjuvant across most chronic and infectious respiratory conditions, with limited evidence only in asthma and interstitial lung diseases.
